# Spot urine protein/creatinine ratio as a reliable estimate of 24-hour proteinuria in patients with immunoglobulin A nephropathy, but not membranous nephropathy

**DOI:** 10.1186/s12882-019-1486-8

**Published:** 2019-08-06

**Authors:** Seiji Kobayashi, Hoichi Amano, Hiroyuki Terawaki, Makoto Ogura, Yoshindo Kawaguchi, Takashi Yokoo

**Affiliations:** 1 Division of Nephrology and Hypertension, Department of Internal Medicine, The Jikei University School of Medicine, Tokyo, Japan; 2 Department of Allergy and Rheumatology, Nippon Medical School Graduate School of Medicine, Tokyo, Japan; 3 Graduate School of Public Health, Teikyo University, Tokyo, Japan; 4 Department of Internal Medicine, Nephrology Teikyo University School of Medicine Teikyo University Chiba Medical Center, Ichihara, Chiba Japan

**Keywords:** 24-h urine, Urine protein excretion, Proteinuria, Nephrotic syndrome, Minimal change disease, Immunoglobulin a nephropathy

## Abstract

**Background:**

Proteinuria is known to be associated with both kidney function deterioration and cardiovascular diseases. While proteinuria estimation from 24-h urine samples has traditionally been considered as the standard method for assessment of the degree of urinary protein excretion, sample collection is associated with several technical problems such as inaccurate collection and the potential spread of drug-resistant pathogens. Therefore, the spot urine protein/creatinine ratio (PCR) assessment is currently recommended as an alternative. While the utility of PCR has been validated, studies on the association between spot urine PCR and 24-h proteinuria (24HP) in patients with chronic glomerular nephritis (CGN) and nephrotic syndrome (NS) are limited. This study aimed to evaluate whether an estimated result from a spot urine PCR could sufficiently approximate the daily urine protein excretion amount from a 24-h urine sample in patients with immunoglobulin A nephropathy (IgAN), minimal change disease (MCD), and membranous nephropathy– nephrotic syndrome (MN-NS).

**Methods:**

The study participants included 161 patients with IgAN, MCD, or MGN-NS at the Jikei University Kashiwa Hospital and Kanagawa Prefecture Shiomidai Hospital. The correlation between spot urine PCR and a 24-h urine protein was investigated using linear regression analysis with Spearman’s correlation (r) coefficient and intraclass correlation coefficient (ICC).

**Results:**

While high correlation coefficients (r = 0.86, *P* < 0.001) and substantial agreement (ICC: 0.806, P < 0.001) were observed in patients with IgAN, similar correlations were not observed in patients with MCD or MN-NS. In the patients with MCD, r was 0.53 (P < 0.001), which signified a slight correlation, and in the patients with MN-NS, r was 0.289 (*P* = 0.17), which was not statistically significant.

**Conclusions:**

This study revealed that spot urine PCR is a reliable estimate of 24HP value in patients with IgAN. In contrast, there is a considerable difference between the daily urine protein excretion amount based on a 24-h urine sample and that which is calculated from spot urine PCR in patients with NS.

## Background

Accumulating evidence shows that assessment of proteinuria plays an important role in the diagnosis of kidney disease and in monitoring disease activity [1–3]. In addition, increase in proteinuria is not only a risk factor for kidney function decline but also for cardiovascular disease [4, 5]. Therefore, assessment of the actual amount of proteinuria is crucial.

The standard method to assess proteinuria is the protein content of an accurately collected 24-h urine sample [6]. However, some problems are encountered in collecting such samples. First, such collection is cumbersome and frequently inaccurate owing to collection error [7]. Second, collection of 24-h urine in the hospital can cause spread of some species of bacteria, such as multidrug-resistant *Pseudomonas aeruginosa*, which causes urinary tract infection [8, 9].

The Japanese Society of Nephrology or Kidney Disease Outcomes Quality Initiative (KDOQI) recommends substituting a spot urine protein/creatinine ratio (PCR) assessment for 24-h urine testing [7, 10]. Several studies validating spot urine PCR in patients with chronic kidney diseases (CKD) or normal kidney function have been conducted [5, 11, 12]. However, studies on the association between a spot urine PCR and 24-h urine protein (24HP) in patients with chronic glomerular nephritis (CGN) and nephrotic syndrome (NS) are limited.

This study aimed to evaluate the correlation and agreement between spot urine PCR with urine protein excretion measured by 24 h urinary collection in patients with CGN and NS.

## Methods

### Patient population

The study participants included 161 patients with immunoglobulin A nephropathy (IgAN), minimal change disease (MCD), or membranous nephropathy –nephrotic syndrome (MN-NS) and who were diagnosed through kidney biopsy at the Jikei University Kashiwa Hospital and Kanagawa Prefecture Shiomidai Hospital between 2008 and 2015.

Patients with diabetes mellitus or taking renin-angiotensin-aldosterone system inhibitors were excluded, because the amount of proteinuria could be influenced by the treatments of underlying disease.

### Data collection

#### Clinical and laboratory information

Data on baseline demographics and clinical and laboratory data were reviewed prior to renal biopsy. Age, sex, body weight, body mass index (BMI), and body surface area were obtained from each medical chart. Serum creatinine (Cr) levels were measured by routine methods (enzyme assay; CRE-II, Kainos Laboratory Inc., Tokyo, Japan) at each hospital. Estimated glomerular filtration rate (eGFR) is expressed using the following formula: eGFR (mL/min/1.73 m^2^) =194 × Cr − 1.094age^-0.287^ (× 0.739, if female) prepared by the Japanese Society of Nephrology [3].

All participants were given explanations on the use of the device for proportionally collectable urine at a rate of 1/50 (Urine-MateP^R^, Sumitomo Bekelite, Tokyo, Japan) for each voiding for 24 h. Casual spot urine was obtained in the morning of the same day after a patient carried out collection of 24-h urine. Urinary concentration of protein and Cr were measured by Japan Electron Optics Laboratory autoanalyzer, Tokyo, Japan.

### Histopathological diagnosis

All kidney tissue specimens were obtained via percutaneous needle biopsies. All specimens were examined using light microscopy; immunohistochemistry, including staining for IgG, IgA, IgM, C3, and C1q; and electron microscopy.

IgAN was diagnosed based on light microscopic findings of mesangial proliferative changes, immunofluorescence findings of mesangial IgA and C3 deposition, and electron microscopic findings of electron-dense deposits in the mesangial area [13].

No abnormalities of kidney tissue on light microscopy were found in patients with MCD. However, podocyte damage was found on electron microscopy [14].

In MN-NS, diagnostic features included capillary wall thickening, normal cellularity, IgG and C3 found along the capillary walls upon immunofluorescence, and subepithelial deposits visible on electron microscopy [15].

There were no patients with the pathological features of diabetic nephropathy.

### Ethical committee

The study protocol was approved by the ethical committee of the Jikei University Kashiwa Hospital and Kanagawa Prefecture Shiomidai Hospital. Informed consent was not obtained from an individual patient, because their laboratory data used in this study were extracted from routine examinations files and analyzed retrospectively. However, we posted the research content at each hospital and given the opportunity to refuse to participate in this research.

### Statistical analysis

Data are expressed as numbers (%), means (standard deviation) or median (25th percentile, 75th percentile).

Based on previous studies, we considered a 500 mg difference in proteinuria as clinically meaningful when estimating 24HP using spot urine PCR. We calculated that with a sample of 126 patients, the study would have 80% power to detect a 500 mg mean difference in proteinuria, with a type 1 error of 5%. For power analysis, we used a standard division of 750 mg [16].

The difference of the mean value between the three groups was assessed using analysis of variance (ANOVA). Categorical data were tested by the chi-square test. Variables without normality of data distribution were tested by the Mann-Whiney U test. The correlation between spot urine PCR and a 24HP was investigated using linear regression analysis with Spearman’s correlation coefficient (r) and the agreement was assessed by intraclass correlation coefficient (ICC). The Cohen’s kappa coefficient (κ) was also calculated to evaluate the consistency between spot urine PCR and a 24 HP, in which spot urine PCR (g/gCre) and a 24 HP (g/day) were classified as ≤0.5, 0.5–1.0, 1.0–3.5 and ≥ 3.5.

In general, the following regression coefficients were obtained: 0.7–1.0, for strong correlation; 0.4–0.7, for slight correlation; 0.2–0.4, for weak correlation; 0–0.2, for almost no correlation. In the case of ICC evaluation score, 0.20 was considered slight, 0.21–0.40, fairer; 0.41–0.60, moderate; 0.61–0.80, substantial; and 0.81, almost perfect. And κ was used to define the level of agreement obtained: κ < 0, poor agreement; κ = 0–0.20, minimal agreement; κ = 0.21–0.40, fair agreement; 0.41–0.60, moderate agreement; 0.61–0.80, substantial agreement; and 0.81–1, almost perfect or perfect agreement.

The 24HP and mean differences between the results of 24HP and spot urine PCR were assessed using Bland–Altman analysis ((A) all patients, (B) IgAN, (C) MCD, and (D) MN-NS). The limits of agreement for each comparison are indicated by the average difference ± 1.96 standard deviations of the difference.

A two-sided *p* value of 0.05 was considered statistically significant. Statistical analysis was performed using the IBM SPSS version 18 software (IBM Corp., Armonk, NY, USA).

## Results

The patients’ characteristics are shown in Table 1. In this study, 84, 53, and 24 patients had IgAN, MCD, and MN-NS, respectively. The mean age was 44.9 ± 18.9 years, and 56.5% of the patients were male. The patients with MN-NS were older than the others.

**Table 1:** Patients’ characteristics

	All cases *N* = 161	IgAN *N* = 84	MCD *N* = 53	MGN-NS *N* = 24	*P* values (by ANOVA)
Age, years	44.9 ± 18.9	40.0 ± 15.9	43.5 ± 20.3	65.0 ± 10.8	< 0.001
Male, n (%)	91 (56.5)	43 (51.2)	31 (58.5)	17 (70.8)	0.217
Creatinine, mg/dL	1.10 ± 0.87	0.95 ± 0.40	1.32 ± 1.30	1.11 ± 0.84	0.051
eGFR, mL/min/1,73 m^2^	69.8 ± 27.6	72.2 ± 24.9	69.1 ± 32.5	62.7 ± 24.5	0.326
Spot urinary protein, mg/dL	749 ± 1148	129 ± 152	1506 ± 1315	1249 ± 1483	< 0.001
Spot urinary creatinine, mg/dL	151 ± 125	115 ± 93	207 ± 155	153 ± 110	< 0.001
24-h urinary creatinine, g/day	1.17 ± 0.47	1.23 ± 0.4	1.16 ± 0.49	1.00 ± 0.29	0.1
24-h urinary protein, mg/dL	466 ± 704	84 ± 92	1028 ± 867	560 ± 668	< 0.001
Daily urinary protein, g/day	4.32 ± 4.51	1.22 ± 1.24	8.44 ± 4.88	6.00 ± 2.29	< 0.001
24-h urine volume, mL/day	1502 ± 769	1655 ± 711	1242 ± 809	1537 ± 750	0.008

Data are expressed as means ± standard deviation or numbers (%). *IgAN* denotes immunoglobulin A nephropathy, *MCD* minimal change disease, *MGN-NS* Membranous glomerulonephritis with nephrotic syndrome, *ANOVA* analysis of variance, *eGFR* estimated glomerular filtration rate

There were no body weight, BMI, and body surface area differences among each group.

Among all patients, the mean serum Cr was 1.10 ± 0.87 mg/dL, and eGFR was 69.8 ± 27.6 mL/min/1.73 m^2^. The patients with MCD had a higher level of serum Cr and lower level of eGFR than the others. There was no 24-h urinary creatinine differences among each group (*P* = 0.06). The median 24HP value was 3.64 g/day, and the patients with MCD and MN-NS had higher levels of 24HP than the patients with IgAN. The median spot PCR was 2.99, and the value of spot PCR in patients with MCD and MN-NS was higher than that in patients with IgAN.

**Fig. 1:**
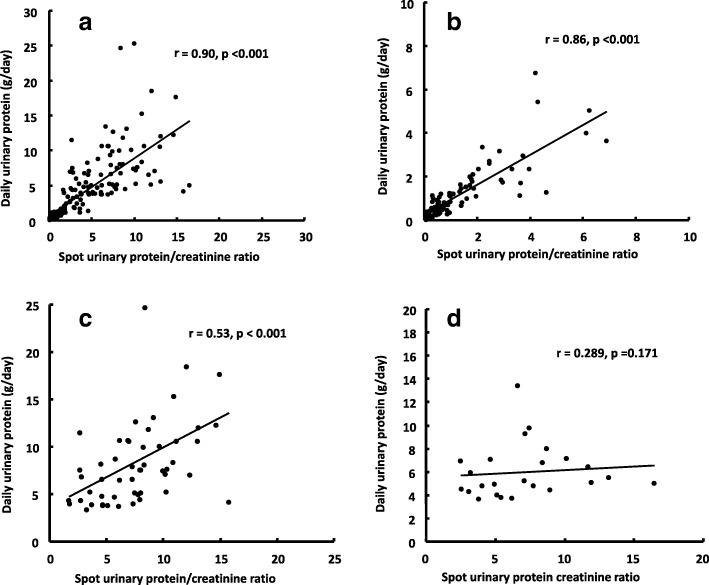
Correlation between spot urinary protein creatinine ratio and daily urinary protein. **a**: All cases (*n* = 161). There is a substantial correlation in all cases (r = 0.9). **b**: IgAN cases (*n* = 84). A strong correlation is recognized in IgAN cases (r = 0.86). **c**: MCD cases (*n* = 53). A slight correlation is found in MCD cases (r = 0.53). **d**: MN-NS cases (*n* = 24). No significant correlation is found in MN-NS cases (r = 0.289)

A strong correlation between 24HP and spot urine PCR in all cases (r = 0.9, *P* < 0.001) and substantial agreement in all cases (ICC: 0.73, 95% confidence interval (95% CI): 0.649–0.795, *P* < 0.001) were found as shown in Table 2. In patients with IgAN, a strong correlation (r = 0.86, *P* < 0.001) and substantial agreement (ICC = 0.806, 95% CI: 0.713–0.871, *P* < 0.001) were found in all cases (Table 2, Fig. 1b). However, in the patients with MCD, r was 0.53 (*P* < 0.001), which was considered as slight correlation, and ICC was 0.42 (95% CI: 0.174–0.617, *P* = 0.001), which was considered to have moderate agreement (Table 2, Fig. 1c). In the patients with MN-NS, r was 0.289 (*P* = 0.17), and ICC was 0.08 (95% CI: − 0.306–0.454, *P* = 0.346), which were not statistically significant (Table 2, Fig. 1d).

**Table 2:** Correlation between spot urinary protein creatinine ratio and 24-h proteinuria

	All cases *N* = 161	IgAN *N* = 84	MCD *N* = 53	MGN-NS *N* = 24
Pearson’s correlation coefficient (r)	0.734	0.827	0.628	0.092
*P* values	<0.001	<0.001	0.001	0.670
Intraclass correlation coefficient (ICC)	0.730	0.806	0.420	0.080
(95% confidential interval)	(0.649–0.795)	(0.713–0.871)	(0.174–0.617)	(- 0.306–0.454)
*P* values	<0.001	<0.001	0.001	0.346

*IgAN* denotes immunoglobulin A nephropathy, *MCD* minimal change disease, *MGN-NS* Membranous glomerulonephritis with nephrotic syndrome

Same trend was also ensured by method agreement analysis employing κ coefficient. While among all patients, the substantial agreement was recognized (κ: 0.63, *P* < 0.001), among the patients with IgAN, κ was 0.51 (*P* < 0.001) which was considered to have a moderate agreement. However, in the case of patients with MCD, κ was 0.22 (*P* = 0.01), which was considered to have an only fair agreement and in the case of patients with MN-NS, κ was − 0.07 (*P* = 0.65), which was considered to have no agreement.

**Fig. 2:**
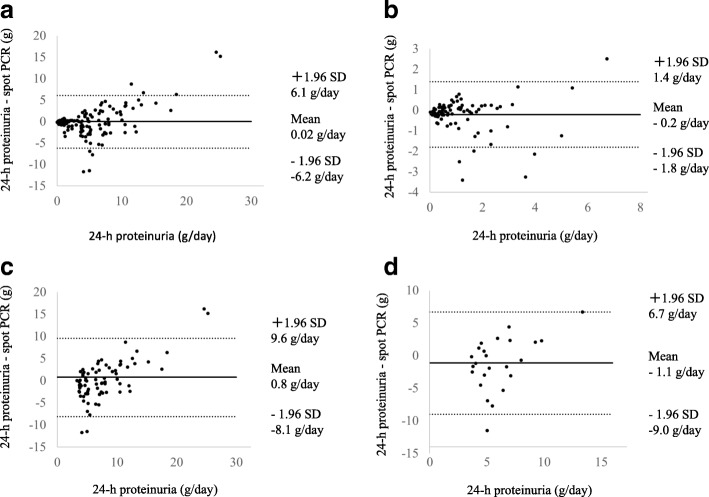
Bland-Altman analysis of the difference between 24-h urinary protein measured by spot urine PCR of all patients (**a**), IgAN (**b**), MCD (**c**), and MN-NS (**d**)

Bland-Altman analysis investigating the association between 24HP and difference between a 24HP and spot urine PCR are shown in Fig. 2a-d. Significant bias was not observed in patients with IgAN. The higher the average volume of proteinuria, the higher the difference between the measured and estimated results on spot urine PCR assessment.

## Discussion

Measurement of urinary protein excretion is a widely accepted method in the detection, diagnosis, and management of people considered to be at risk for developing kidney disease and has been advocated as part of a regular check-up in such individuals [1–3, 17, 18]. Recently, spot urine PCR has been used as a surrogate of the measurement of 24HP in the clinical practice. However, in patients with CGN and NS, the correlation and agreement between spot PCR and 24HP have not been fully elucidated [16, 19, 20]. This study aimed to evaluate the difference of daily urine protein excretion measured by 24HP and spot urine PCR in patients with CGN and NS. Our study showed that in IgAN, spot urine PCR had a strong correlation with 24HP value. However, similar results were not found in both MCD and MGN-NS.

The major finding of our study is that in patients with IgAN, a strong correlation was found between spot urine PCR and 24HP value. Several previous studies showed this correlation in patients with CKD or normal kidney function [6, 11, 21, 22]. However, only a few studies focused on IgAN to investigate the association [23, 24]. Among patients with glomerulonephritis in this cross-sectional study, high correlation coefficients (r = 0.91, 95% CI: 0.95–0.98) were observed [23]. In addition, in a cohort study of 182 selected patients with primary IgAN, a good correlation was found between urine albumin-to-creatinine ratio and 24HP, except those pertaining to CKD stage 5, in patients with IgAN [24]. In the current study, similar results were observed.

Another major finding of our study is that among patients with MCD or MGN-NS, who had nephrotic-range proteinuria, poor or no correlation was found between a spot urine PCR and 24HP value. Until recently, few studies investigating the association between spot urine PCR and 24HP in patients with NS, mainly focused on preeclampsia.

Some studies showed that in patients with nephrotic-range proteinuria, this association was not related [25, 26]. Previous observational studies demonstrated that the random urine albumin/creatinine ratio was a poor predictor for a proteinuria of > 2 g/day in patients with preeclampsia [25]. Another study investigated that among 220 women with preeclampsia, for severe proteinuria, a urine PCR of ≥5000 mg/g had a poor positive predictive value (61.9%) and sensitivity (72.2%) [26], while other few studies have shown that a spot urine PCR and 24HP in patients with high levels of protein in urine are related [27]. The previous study showed that even when a large amount of protein in urine is observed in nephrotic syndrome, the coincidence rate is 89 to 94% [27]. The reason for these inconsistencies needs to be fully elucidated; however, previous results differed with regard to age, race, country, timing of a spot urine, and patients’ diseases.

Several reasons can be considered why a spot urine PCR is reliable for 24HP in patients with IgAN, but unreliable in patients with MCD or MN-NS. Megalin may play an important role in our findings. Megalin, which is known as a low-density lipoprotein-related protein, is one of the large transmembrane proteins expressed on the surface of proximal tubular epithelial cells, where they are central to the endocytic reabsorption of many plasma proteins filtered across the glomerular capillary wall [28–30]. Owing to reabsorption of protein in urine by megalin, patients with normal kidney function have very limited amount of proteins in urine. However, in nephrotic-range diseases such as MCD or MN-NS, the mechanism behind these observations remains unclear. Previous studies showed that deficiency of megalin is likely to be associated with the development of proteinuria/albuminuria and increased urinary megalin excretion is associated with tissue damage [31–33]. Ogasawara et al. suggested that urinary C-megalin levels were significantly high in patients with normoalbuminuria, were elevated in line with increased albuminuria, and reduced kidney function [33]. In nephrotic-range disease, we hypothesized that the causes why spot urine PCR was not associated with 24HP were as follows: In nephrotic syndrome, kidney function may decrease because of causes such as dehydration, and increase of sodium reabsorption [14]; as a result, megalin function declines, affecting its role in the reabsorption of proteins in urine (2). In excessive protein excretion, variations occurred in megalin reabsorption. Therefore, the fluctuations in spot urine PCR alone were small; however, as the 24HP increased, the fluctuations in spot urine PCR also increased [27]. We demonstrated that when nephrotic-range proteinuria was present, there was less actual predictive value between a spot urine PCR and 24HP. Future studies are warranted to investigate the association according to the amount of proteinuria difference.

This study had several limitations. First, the findings cannot be generalized to other ethnic or age groups, because the subjects were only eligible patients in the core hospitals. However, because the core hospitals included a university hospital and a prefectural hospital in different prefectures in Japan, our study may be validated. Second, we studied only the correlation of one sample both from 24HP value and spot urine PCR. Moreover, our results were not demonstrated in other populations; therefore, external validity might not be proved. Third, there was also the possibility of sampling error when collecting the 24-h urine sample. Generally, it is said that 24 h urine Cr excretion is 1 g/day. In each group, 24 h urine Cr excretion was more 1 g/day and there was no difference among each group. Therefore, the validity of collecting 24-h urine is considered to be reasonable.

## Conclusions

We demonstrated the strong correlation with 24HP value and a spot urine PCR in patients with IgAN. A spot urine PCR is reliable with 24HP in patients with IgAN. In contrast, in patients with MCD and MN-NS, clinicians should understand that difference should be considered in the calculation of the amount of daily urinary protein excretion between 24HP and a spot urine PCR.

## Data Availability

The datasets generated and analyzed during the study are not publicly available due the terms of consent to which the participants agreed, but the datasets generated and/or analyzed during the current study are available upon request from the corresponding author.

## Abbreviations

24HP: 24-h proteinuria
ICC: Intraclass correlation coefficient
IgAN: Immunoglobulin A nephropathy
MCD: Minimal change disease
MGN-NS: Membranous glomerulonephritis with nephrotic syndrome
PCR: Protein/creatinine ratio
r: Spearman’s correlation
